# Supplementary material for: Lonicera Caerulea Juice Alleviates Alcoholic Liver Disease by Regulating Intestinal Flora and the FXR-FGF15 Signaling Pathway
Source: Nutrients. 2023 Sep 17;15(18):4025. doi: 10.3390/nu15184025 (PMC10534805; doi:10.3390/nu15184025)
Supplement: Supplementary file 1 [file nutrients-15-04025-s001.zip › nutrients-2566294-supplementary.pdf]

Supplementary Table S1. Quantitative RT-PCR primer design.

Target Gene	Sequences (50–30)	Reference
Gapdh	F: GTGCCGCCTGGAGAAACC<br>R: GGTGGAAGAGTGGGAGTTGC	[1]
β-actin	F: TGTCCACCTTCCAGCAGATGT<br>R: AGCTCAGTAACAGTCCGCCTAGA	[2]
MUC2	F: ACGTGTCATATTTGCACCTCT<br>R: TCAACATTGAGAGTGCCAACT	[3]
MUC4	F: TCTTTCTGTCTCAACTGTTGAATCAGA<br>R: CGTGGCCAGGATGTCAAAC	[4]
ZO-1	F: GCCGCTAAGAGCACAGCAA<br>R: TCCCCACTCTGAAAATGAGGA	[5]
Cldn1	F: ACGGTCTTTGCACTTTGGTC<br>R: GGGAGAGGAGAAGCACAGTT	[5]
FGF15	F: GGCAAGATATACGGGCTGAT<br>R: TCCATTTCCTCCCTGAAGGT	[6]
FXR	F: GGCAGAATCTGGATTTGGAATCG<br>R: GCTGAACTTGAGGAAACGGG	[7]
FGFR4	F: TTGGCCCTGTTGAGCATCTTT<br>R: GCCCTCTTTGTACCAGTGACG	[8]
CYP7A1	F: GGGATTGCTGTGGTAGTGAGC<br>R: GGTATGGAATCAACCCGTTGTC	[7]
AMPK	F: TGAAGATCGGCCACTACATC<br>R: TTGCCCACCTTCACTTTCC	[9]
CPT1b	F: CATGTATCGCCGCAAACTGG<br>R: CCTGGGATGCGTGTAGTGTT	[10]
PPARα	F: TGGGGATGAAGAGGGCTGAG<br>R: GGGGACTGCCGTTGTCTGT	[11]

Supplementary Table S2. Phenolic components in LCJ.

Assays	Content
Total phenol	18.00 ± 0.01 mg/mL
Cyanidin-3-glucoside	394.57 ± 0.06 mg/100 mL
Cyanidin-3-galactoside	275.48 ± 0.22 mg/100 mL
Chlorogenic	153.56 ± 0.17 mg/100 mL
Quercetin	96.44 ± 0.02 mg/100 mL
Isochlorogenic acid C	16.77 ± 0.31 mg/100 mL
Caffeic acid	15.18 ± 0.42 mg/100 m
Phlorizin	8.50 ± 0.33 mg/100 mL
Catechin	7.92 ± 0.16 mg/100 mL
Aromadendrin	1.97 ± 0.07 mg/100 mL
Neodiosmin	1.13 ± 0.06 mg/100 mL
Naringenin	0.96 ± 0.09 mg/100 mL
Procyanidin B2	0.67 ± 0.05 mg/100 mL
Epicatechin	0.37 ± 0.05 mg/100 mL
Astragalin	0.27 ± 0.07 mg/100 mL
7-Hydroxycoumarin	0.17 ± 0.03 mg/100 mL
Cynarin	0.07 ± 0.06 mg/100 mL

Supplementary Table S3. Comparison Table of English Abbreviations

acronym	Full Name
ALD	Alcoholic liver damage
LCJ	*Lonicera caerulea* juice
LC	*Lonicera caerulea*
TG	Triglyceride
TC	Total cholesterol
CG	Control group
AG	Alcohol group
LG	Low-dose LCJ group (total phenol intake: 150 mg/kg)
HG	High-dose LCJ group (total phenol intake: 300 mg/kg)
SCG	Small intestine in the control group
SAG	Small intestine in the alcohol group
SHG	Small intestine in the high-dose LCJ group
LCG	Large intestine in the control group
LAG	Large intestine in the alcohol group
LHG	Large intestine in the high-dose LCJ group
DADA2	Divisive amplicon denoising algorithm
SCFAs	Short-chain fatty acids
FXR	Farnesoid X receptor
FGF15	Fibroblast growth factor 15
FGFR4	Fibroblast growth factor receptor 4
CYP7A1	Cholesterol 7α-hydroxylase
CPT1b	Recombinan Carnitine Palmitoyltransferase 1b
LPS	Lipopolysaccharide
MUC2	Mucin2
ZO-1	Zona occludens 1
AMPK	Adenosine 5′-monophosphate (AMP)-activated protein kinase
PPAR α	Peroxisome proliferator-activated receptor
MUC4	Mucin4
TBA	Total bile acid
qPCR	Quantitative Real-time PCR
HE	Hematoxylin-eosin

ALT	Alanine aminotransferase
AST	Aspartate aminotransferase
AB-PAS	Eriodic acid Schiff and Alcian blue
WB	Western blotting
TβMCA	Tauro-β-muricholic acid
LCA	Lithocholic acid
UDCA	Ursodeoxycholic acid
BSH	Bile salt hydrolase
CDCA	Chenodeoxycholic Acid
DCA	Deoxycholic acid
CA	Cholic acid
TDCA	Tauroursodeoxycholic acid
TCA	Taurocholate acid
GCDCA	Glycochenodeoxycholic acid

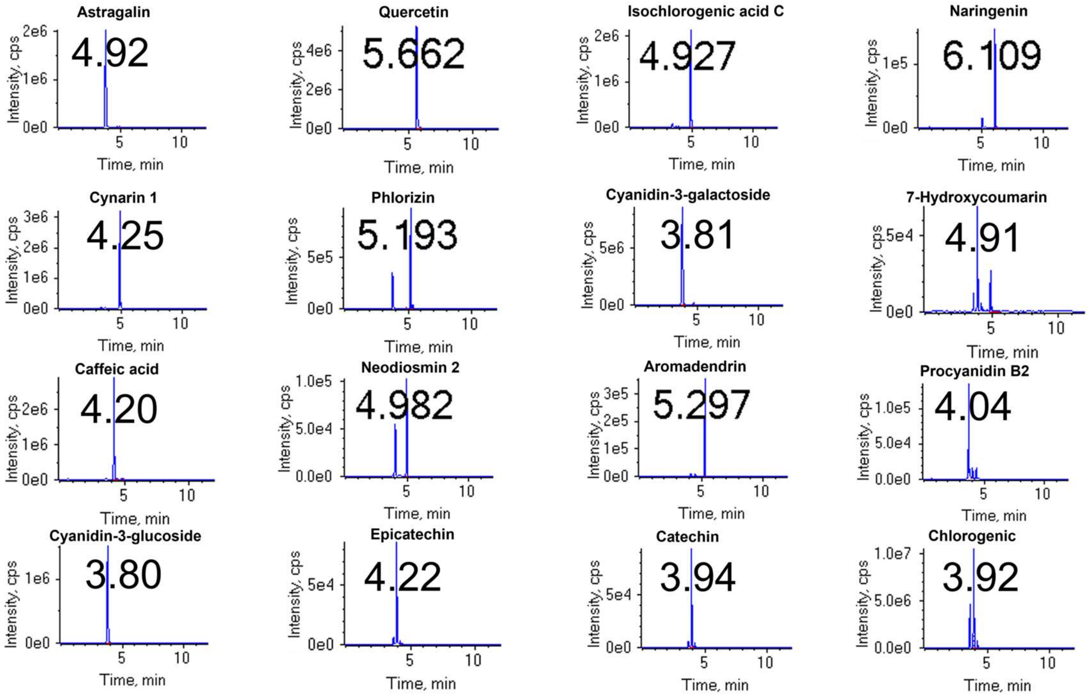

Supplementary Figure S1. Total chromatograms of 16 types of phenols in LCJ.

1. Li, C.; Yu, S.; Li, X.; Cao, Y.; Li, M.; Ji, G.; Zhang, L. Medicinal Formula Huazhi-Rougan Attenuates Non-Alcoholic Steatohepatitis Through Enhancing Fecal Bile Acid Excretion in Mice. *Front Pharmacol* **2022**, *13*, 833414, doi:10.3389/fphar.2022.833414.
2. Li, J.; Zhang, L.; Li, Y.; Wu, Y.; Wu, T.; Feng, H.; Xu, Z.; Liu, Y.; Ruan, Z.; Zhou, S. Puerarin improves intestinal barrier function through enhancing goblet cells and

mucus barrier. *Journal of Functional Foods* **2020**, *75*, 104246, doi:https://doi.org/10.1016/j.jff.2020.104246.
3. Lu, F.; Li, Y.; Zhou, B.; Guo, Q.; Zhang, Y. Early-life supplementation of grape polyphenol extract promotes polyphenol absorption and modulates the intestinal microbiota in association with the increase in mRNA expression of the key intestinal barrier genes. *Food & Function* **2021**, *12*, 602-613, doi:10.1039/D0FO02231D.
4. Wang, H.H.; Afdhal, N.H.; Gendler, S.J.; Wang, D.Q.H. Targeted disruption of the murine mucin gene 1 decreases susceptibility to cholesterol gallstone formation1. *Journal of Lipid Research* **2004**, *45*, 438-447, doi:https://doi.org/10.1194/jlr.M300468-JLR200.
5. Jiang, S.; Xue, D.; Zhang, M.; Li, Q.; Liu, H.; Zhao, D.; Zhou, G.; Li, C. Myoglobin diet affected the colonic mucus layer and barrier by increasing the abundance of several beneficial gut bacteria. *Food Funct* **2022**, doi:10.1039/d2fo01799g.
6. Danjo, T.; Eiraku, M.; Muguruma, K.; Watanabe, K.; Kawada, M.; Yanagawa, Y.; Rubenstein, J.L.; Sasai, Y. Subregional specification of embryonic stem cell-derived ventral telencephalic tissues by timed and combinatory treatment with extrinsic signals. *J Neurosci* **2011**, *31*, 1919-1933, doi:10.1523/jneurosci.5128-10.2011.
7. Sun, C.C.; Zhang, C.Y.; Duan, J.X.; Guan, X.X.; Yang, H.H.; Jiang, H.L.; Hammock, B.D.; Hwang, S.H.; Zhou, Y.; Guan, C.X.; et al. PTUPB ameliorates high-fat diet-induced non-alcoholic fatty liver disease via inhibiting NLRP3 inflammasome activation in mice. *Biochem Biophys Res Commun* **2020**, *523*, 1020-1026, doi:10.1016/j.bbrc.2019.12.131.
8. Zhao, Y.; Meng, C.; Wang, Y.; Huang, H.; Liu, W.; Zhang, J.-F.; Zhao, H.; Feng, B.; Leung, P.; Xia, Y. Interleukin (IL)-1β inhibits β-Klotho expression and FGF19 signaling in hepatocytes. *American journal of physiology. Endocrinology and metabolism* **2015**, *310*, ajpendo.00356.02015, doi:10.1152/ajpendo.00356.2015.
9. Zhang, L.; Duan, Y.; Guo, Q.; Wang, W.; Li, F. A selectively suppressing amino acid transporter: Sodium-coupled neutral amino acid transporter 2 inhibits cell growth and mammalian target of rapamycin complex 1 pathway in skeletal muscle cells. *Anim Nutr* **2020**, *6*, 513-520, doi:10.1016/j.aninu.2020.03.010.
10. Lee, J.; Narayan, V.P.; Hong, E.Y.; Whang, W.K.; Park, T. Artemisia Iwayomogi Extract Attenuates High-Fat Diet-Induced Hypertriglyceridemia in Mice: Potential Involvement of the Adiponectin-AMPK Pathway and Very Low Density Lipoprotein Assembly in the Liver. *Int J Mol Sci* **2017**, *18*, doi:10.3390/ijms18081762.
11. Bak, E.J.; Kim, J.; Choi, Y.H.; Kim, J.H.; Lee, D.E.; Woo, G.H.; Cha, J.H.; Yoo, Y.J. Wogonin ameliorates hyperglycemia and dyslipidemia via PPARα activation in db/db mice. *Clin Nutr* **2014**, *33*, 156-163, doi:10.1016/j.clnu.2013.03.013.
